# The immune system on the TRAIL of Alzheimer’s disease

**DOI:** 10.1186/s12974-020-01968-1

**Published:** 2020-10-13

**Authors:** Chiara Burgaletto, Antonio Munafò, Giulia Di Benedetto, Cettina De Francisci, Filippo Caraci, Rosaria Di Mauro, Claudio Bucolo, Renato Bernardini, Giuseppina Cantarella

**Affiliations:** 1 Department of Biomedical and Biotechnological Sciences (BIOMETEC), Section of Pharmacology, University of Catania, Via Santa Sofia 97, Catania, Italy; 2 Department of Drug Sciences, University of Catania, Catania, Italy; 3 Oasi Research Institute-IRCCS, Troina, Italy; 4 Clinical Toxicology Unit, University Hospital, University of Catania, Catania, Italy

**Keywords:** Immune response, Neuroinflammation, Proinflammatory cytokines, Regulatory T cells

## Abstract

Alzheimer’s disease (AD) is the most common form of dementia, characterized by progressive degeneration and loss of neurons in specific regions of the central nervous system. Chronic activation of the immune cells resident in the brain, peripheral immune cell trafficking across the blood-brain barrier, and release of inflammatory and neurotoxic factors, appear critical contributors of the neuroinflammatory response that drives the progression of neurodegenerative processes in AD. As the neuro-immune network is impaired in course of AD, this review is aimed to point out the essential supportive role of innate and adaptive immune response either in normal brain as well as in brain recovery from injury. Since a fine-tuning of the immune response appears crucial to ensure proper nervous system functioning, we focused on the role of the TNF superfamily member, TNF-related apoptosis-inducing ligand (TRAIL), which modulates both the innate and adaptive immune response in the pathogenesis of several immunological disorders and, in particular, in AD-related neuroinflammation. We here summarized mounting evidence of potential involvement of TRAIL signaling in AD pathogenesis, with the aim to provide clearer insights about potential novel therapeutic approaches in AD.

## Background

Alzheimer’s disease (AD) is an age-related neurodegenerative disorder with an insidious onset characterized by cerebral atrophy and progressive cognitive decline [1]. The acknowledged neuropathological hallmarks of AD are represented by extracellular senile plaques, composed of amyloid-β (Aβ) peptide and intracellular neurofibrillary tangles (NFTs) generated by hyperphosphorylated protein tau [2].

Growing evidence suggests that the multifactorial pathophysiological mechanisms of AD is not restricted to the neuronal compartment, as relevant role has been attributed to the tight interactions of immunological mechanisms within the brain [3].

Since decades, active research has investigated network connections between the immune system and the nervous system. In fact, it has been described a reciprocal functional control between the immune system and the central nervous system (CNS) [4], a mechanism essential to tissue repair and regeneration as well as removal of damaged tissues and cells [5]. A low-grade peripheral immune/inflammatory response and the basal release of cytokines are needed to maintain brain homeostasis and functional plasticity, including hippocampal-dependent cognitive functions and neurogenesis, suggesting that the systemic immune response exerts a healing role in the CNS [6, 7].

Now, it is a common notion that systemic inflammatory disorders may be associated with cognitive decline [8], and, in fact, chronic inflammation is known to inhibit neuronal functions and contribute to onset and progression of AD [9]. In this line, robust data support the crucial relevance of mediators of the inflammatory/immune response in neurodegeneration, as, for instance, injured neurons release arrays of these molecules, which redundantly sustain neuronal damage and death [10].

Cytokines belonging to the tumor necrosis factor (TNF) superfamily are considered substantial contributors of the accelerated cell death rate which characterizes neurodegenerative processes. Among these, the proapoptotic/proinflammatory cytokine Tumor necrosis factor-related apoptosis-inducing ligand (TRAIL), first discovered as a tumor cell killer, is expressed in macrophages, T lymphocytes, neutrophils, and dendritic cells [11, 12].

TRAIL, which acts through two death receptors referred to as DR4 and DR5, is a potent mediator of prominent neuronal loss induced in both chronic and acute neurodegenerative processes, including those related to Aβ accumulation [13], trauma [14], and brain ischemia [15, 16], consistent with boosted peri-damage neuroinflammation.

Furthermore, sustained TRAIL expression appears related to functional decline in animal models of AD [17]. In fact, its immunoneutralization by means of a monoclonal antibody is associated with a significant rescue of neurons from death [13], reduced accumulation of Aβ and attenuated expression of inflammatory/immune mediators [17], paralleled by a re-balance of both central, and peripheral immune response [18].

In synthesis, TRAIL efficiently sets into motion and sustain neurodegeneration-related neuroinflammation, as its neutralization implies significant attenuation of inflammatory processes [19], corroborating the hypothesis that is represents an important molecular clue to Aβ-dependent neurodegenerative processes, and may thus well be envisioned as a potential candidate target for innovative immunotherapeutic strategies in AD.

### The impact of central and peripheral inflammatory/immune response in Alzheimer’s disease

Neurodegenerative disorders share selective neuronal vulnerability in specific brain regions, which is related to the neuronal responses to detrimental stimuli, such as, for instance, disease-related misfolding proteins, that finally become unsupportive to neurons [20].

In addition to the pathogenetic role of Aβ and tau proteins in AD, recent evidence favors the hypothesis that the immune system plays a pivotal role in the onset and progression of this disease [21].

In fact, neuronal damage in AD is associated with chronic activation of the CNS-resident innate immune cells and increased peripheral leukocyte access across the blood-brain barrier (BBB) [22], consistent with the demonstration of a functional meningeal lymphatic system [23], as well as of a substantial peripheral immunocyte trafficking through the choroid plexus (CP) [24], supporting the notion of a cross-talk system between peripheral and CNS immunocytes.

Moreover, the innate immune system indeed represents the first line of defense against pathogens serving as a link to adaptive T and B cells, by means of antigen presentation processes and transfer of information [25, 26], and in this line, both branches of immune response, adaptive, and innate, may affect the neuroinflammatory process and related progression of neurodegeneration in AD and other CNS disorders [27].

It is well-established that microglia and astrocytes, the predominant innate immune cells in the CNS, are strongly implicated in aberrant molecular pathways that underlie AD pathogenetic alterations [10].

Microglial cells represent the major immunological effector of the innate immune system in the brain and mediate functions such as tissue surveillance, removal of pathogens, and response to injury [28, 29], also contributing to neuronal survival and synaptogenesis [30].

Under resting condition, microglia are characterized by a ramified morphology and a weak antigen-presenting activity, partly due to low level of expression of the major histocompatibility complex (MHC) on its surface [31]. Activated microglia eventually convert to an amoeboid-like morphology which displays upregulated expression of both MHC and co-stimulatory molecules involved in antigen presentation, leading to interactions with peripheral immune cells [32].

**Fig. 1:**
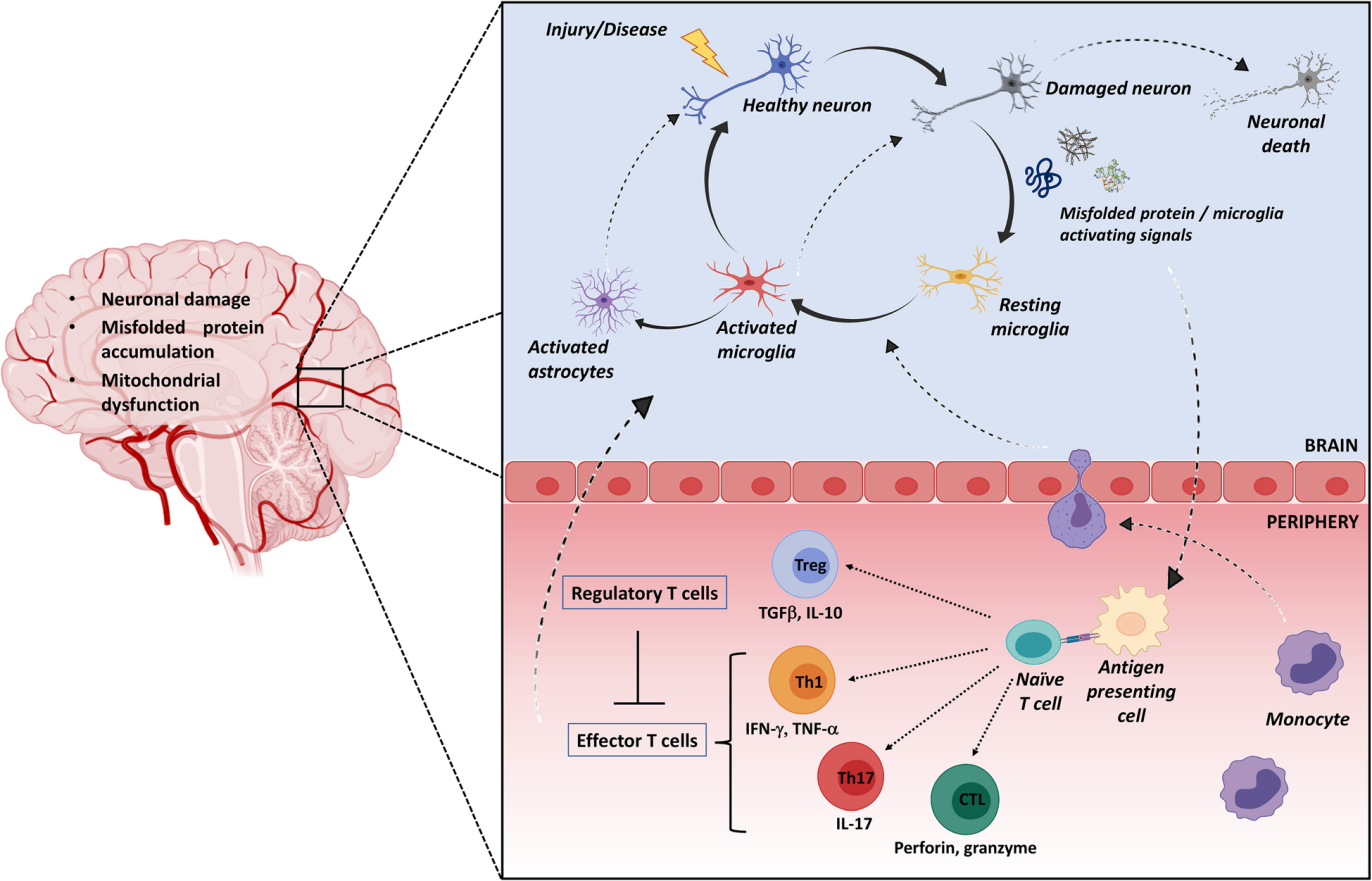
Central and peripheral inflammatory/immune response in neurodegeneration. Upon injury, disease, or inflammation, damaged neurons could release self-antigens or modified proteins that activate resting microglia. Activated microglia responds to these stimuli, by production of proinflammatory cytokines and chemokines, reactive oxygen, and nitrogen species. When such first innate immune-related process is not completely resolutive and the inflammatory stimuli persist, the microglia-mediated mechanisms remain trapped in a vicious cycle, characterized by chronic pro-inflammatory cytokine production linked to a cascade of neurotoxic events leading to neuronal death. Substantial recruitment of monocytes into the AD brain begins when Aβ deposition and associated neuronal damage triggers a local immune response, activating astrocytes and microglia. Activated pro-inflammatory microglia also release astrocyte-activating signals which induce neuroinflammatory astrocytes that, in turn, amplify the neurodegenerative cycle. In addition, misfolded proteins not adequately removed may drain into peripheral lymphoid tissues, wherein they are presented by antigen presenting cells to naïve T cells, thereafter mounting an adaptive immune response against these antigens. Depending upon antigen-presenting cell signals, naïve T cells differentiate into antigen-specific T effector cells (Th1, Th2, Th17, and cytotoxic T lymphocytes (CTL) or regulatory T (Treg) cells). Specifically, Th1 and Th17 cells cross the blood-brain barrier and directly contribute to neuroinflammation through the production of neurotoxic and proinflammatory factors that act on glial cells. Consequently, activated microglia and astrocytes respond by releasing high amounts of chemokines that assist the infiltration of a second wave of effector T cells into the brain. CD8+ CTLs recognize antigen presented by MHC class I on neurons to induce perforin- and/or granzyme-mediated cytolysis. In response to inflammatory events, Treg cells dampen down neuroinflammation and neurodegeneration

Upon injury, disease, or inflammation, healthy neurons may get damage, which in turn causes release of self-antigens or aberrant proteins that activate resting microglia (Fig. 1). In fact, pathogenic stimuli break the delicate balance between neurotoxic and neuroprotective mechanisms, inducing microglial activation, triggering for example, the Toll-like receptors 4 (TLR4) signaling pathway and conversion to the pro-inflammatory phenotype [33–35]. The latter microglial state is characterized, not only by a morphological changes, but also by release of pro-inflammatory molecules, such as interleukin-1 beta (IL-1β), interleukin-6 (IL-6), tumor necrosis factor alpha (TNFα), interferon gamma (IFN-γ), chemokines, as well as reactive oxygen and nitrogen species (ROS/RNS), which promote diapedesis of peripheral leukocytes through the BBB, further contributing to fuel local detrimental inflammatory response [28, 36].

In addition to its pro-inflammatory pattern, microglia can also adopt an alternative activation pathway [37], associated with increased production of anti-inflammatory cytokines and neurotrophic factors to facilitate phagocytosis of cell debris and promote neuronal repair and survival [38, 39].

In the occurrence that innate immune-related processes are not completely resolutive and the inflammatory stimuli persist, microglia-mediated mechanisms are trapped in a vicious cycle, characterized by chronicized release of pro-inflammatory cytokine initiating a cascade of toxic events leading to neuronal death [40].

Although microglia represent the main mediators of brain immune surveillance, under pathological conditions the infiltrating monocytes transiently supplement the brain mononuclear phagocyte compartment (microglia itself) playing a major role in controlling neuropathological events in the CNS [41, 42]. Notably, infiltrated monocytes contribute to tissue repair, inflammation resolution, and production of neurotrophic factors [41].

Astrocytes, the other major innate effector cells in the CNS, contribute to maintainance of CNS homeostasis and sustain neuronal survival through the release of metabolites and neurotrophic factors essential for normal brain functions and organized cognitive activity [43], and they also safeguard BBB structural integrity and permeability, eventually exerting gate-controlled recruitment of peripheral immune cells into the brain parenchyma [44].

Recent work has highlighted the pathophysiological relevance of the microglia-astrocyte crosstalk [45]. In particular, activated microglia releases specific astrocyte-activating signal molecules, such as interleukin-1 alpha (IL-1α), TNFα, and complement component 1q (C1q), all inductors of a neuroinflammatory reactive astrocyte phenotype, which, similarly to activated microglia, highly express many complement components, including MHC class II molecules [46, 47], as well as an array of cytokines and chemokines that act as chemoattractants, crucial for the recruitment of T cells into the CNS [48–50].

In addition, also adaptive immune cells infiltrating the brain parenchyma seem able to support the neuroinflammatory process [51], as demonstrated for B and T lymphocytes, which are endowed with protective functions from pathogens and trigger a fast specific immune response in case of repeated infections due to the same agent [52].

Under conditions of neurodegeneration, high frequencies of T lymphocytes have been found to infiltrate the brain parenchyma, suggesting a critical pathophysiological role [53]. In fact, the chronic neuroinflammatory status associated with neurodegenerative disorders and driven by the main reactive components of the CNS affects the structural integrity and the permeability of BBB, enhancing transmigration of peripheral immune cells into the CNS and diffusion of inflammatory molecules across the BBB [54–56], thus contributing to the development and progression of lesions [57, 58]. Nevertheless, it has been shown that the CP of the blood-cerebrospinal fluid barrier (BCSFB) works mainly as a selective gateway for leukocyte entry, rather than a firm barrier (BBB) for immune surveillance. Schwartz and colleagues proposed CP as a selective and “educative” gate for recruitment of leukocytes to the inflamed CNS parenchyma [40]. This hypothesis is supported by the findings that neutrophils, monocytes, and T cells enter the injured CNS through the BCSFB in response to brain parenchyma damage [59].

T cells can be classified into CD4+T cells, main regulators of the immune response, and CD8+T cells, designated as cytotoxic T cells for their ability to remove damaged and infected cells [60].

Depending either upon specific stimuli, tissue environment and antigen-presenting cell signaling, naïve CD4+ T cells (Th0) differentiate into antigen-specific T effector including T-helper1 (Th1), T-helper2 (Th2) and T-helper17 (Th17) cells, as well as cytotoxic T lymphocytes (CTLs), or regulatory T cells (Tregs). While Th1 and Th17 cells, which are overactivated in neurodegenerative disorders [61], directly contribute to neuroinflammation through the release of pro-inflammatory cytokines (IFNγ, TNFα, and IL-17) and other inflammatory mediators, Th2 cells, produce anti-inflammatory cytokines (e.g., IL-4), and, for this reason, they have been considered for development of potential intervention strategies [62]. Both Th1 and Th2 cells, are essential for the maintenance of a healthy CNS environment, as an altered Th1/Th2 ratio has been regarded as a causative event in neurodegeneration [63].

Moreover, also antigen-specific CD8+ CTLs have been shown to participate to the pathophysiology of chronic inflammatory disorders includeed those related to neurodegeneration, through production of cytolysis mediators such as perforins and granzymes [64, 65].

Another cell subset, Treg cells have been shown to dampen down neuroinflammation by inhibiting antigen presentation, and upregulating glial neurotrophic factors [66]. As immunoregulatory cells, Tregs release anti-inflammatory factors, such as interleukin-10 (IL-10), and transforming growth factor beta (TGFβ), that suppress activation of effector T lymphocytes, assuming a key role in the development and maintenance of immune tolerance [67].

Because of their immunosuppressive properties, Treg cells, extensively studied in autoimmune disorders [68], actually represent potential elements for improvement of the outcome in neurodegenerative disorders [69, 70].

Recently, the possibility of a dual role of Tregs in the progression of AD has been object of debate. In this condition, Tregs may have a beneficial role at early disease stages, restraining detrimental gliosis, promoting beneficial activation of microglia, and allowing leukocyte re-trafficking through CP [71].

A deficit of TGF-β1, the main cytokine produced by Tregs, can critically contribute to neuroinflammation in AD brain [72, 73]. Additional preclinical studies in experimental models of AD are needed to understand whether Treg cells might exert neuroprotective effects in an early phase of the amyloid-related neurodegeneration by rescue of TGF-β1.

On the other hand, at later disease stages, Tregs appear to take over a detrimental function, by altering CP function and reducing the recruitment of inflammation-resolving leucocytes to CNS [74].

### TRAIL: a potent, pleiotropic fine-tuning effector of the immune response

TRAIL, also known as TNFSF10, is a pleiotropic cytokine belonging to the TNF superfamily, involved in many peripheral and CNS functions, including cell death signaling pathway, immune response, and inflammation [75].

TRAIL can be detected as a soluble and type II transmembrane protein [76, 77]. The homotrimeric and biologically active form is able to interact with a complex system of receptors with different signaling outcomes, from pro-apoptotic to prosurvival/proliferative effects [78, 79].

In humans, TRAIL binds two death-inducing receptors, DR4/TRAIL-R1 and DR5/TRAIL-R2, which contain a functional intracellular death domain, and two transmembrane decoy receptors (DcRs), DcR1/TRAIL-R3 and DcR2/TRAIL-R4, which downregulate the activity of the former receptors by sequestration of the bioactive ligand [80]. Finally, TRAIL has also been shown to bind with very low affinity to osteoprotegerin (OPG), a secreted member of the TNF receptor family involved with the regulation of bone turnover, which acts as a soluble neutralizing receptor [81, 82]. Unlike humans, mice express only three TRAIL receptors: DR5, DcR1, and DcR2 [83, 84].

Two TRAIL-activated death pathway have been identified: an extrinsic pathway, linked to caspase-8 activation, through the recruitment of the adaptor molecule Fas-associated death domain protein (FADD), and an intrinsic mitochondrial pathway in which effector caspases are activated after a BH3 interacting-domain (Bid)-mediated signaling cascade causing mitochondrial outer membrane permeabilization, and the release of cytochrome c which promotes the formation of multimeric complex called “apoptosome” [85].

Several studies suggest the existence of a crosstalk between the two pathways, as demonstrated by the evidence that Bid is cleaved by active caspase-8 [86].

Since its discovery, TRAIL has been extensively studied in the cancer area because of its ability to induce selective apoptosis in a wide variety of tumor cell lines [87]. While the latter has long represented the best characterized function of TRAIL, increasing evidence suggest that TRAIL mediates several alternative functions in normal cells [88]. In fact, TRAIL can stimulate also prosurvival pathways, through factors such as nuclear factor 휅B (NF-휅B) and Akt [89]. In addition, TRAIL promotes proliferation and migration of endothelial cells, suggesting its role in endothelial cell physiology and in the pathophysiology of the vascular system [90, 91].

**Fig. 2:**
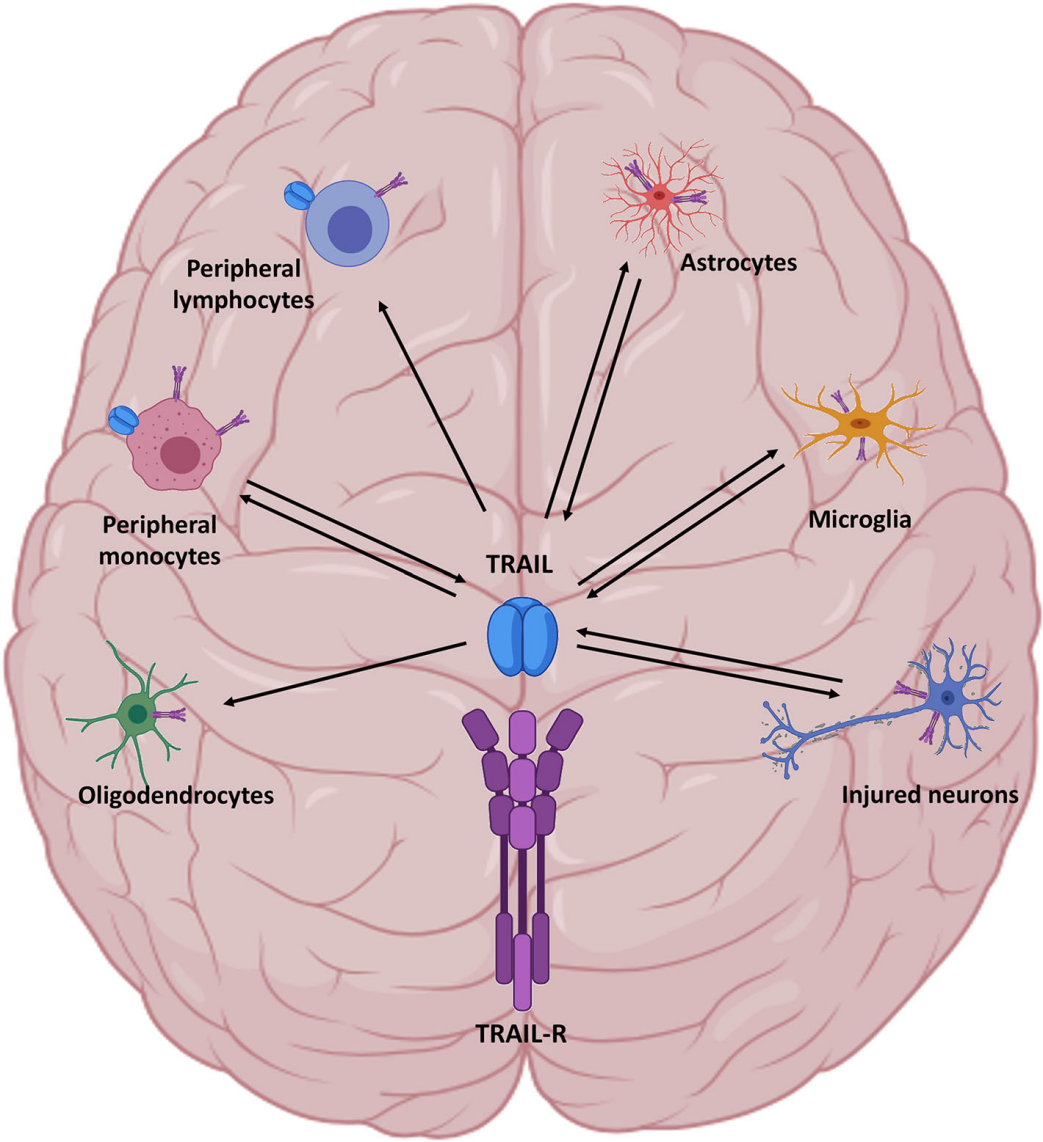
Fine-tuning of immune response by TRAIL in the brain. Under brain inflammatory conditions, TRAIL is abundantly released by activated glia, infiltrated peripheral monocytes and injured neurons. TRAIL acts as a potential death signal by interacting with its receptors expressed in neurons, microglia, monocytes, lymphocytes, astrocytes, and oligodendrocytes

Among others, a major role of TRAIL appears related to the fine-co-tuning of the immune response in the CNS (Fig. 2).

TRAIL is not costitutively expressed in the normal brain, which, instead, expresses its receptors [92], while under inflammatory brain conditions, TRAIL is abundantly released by activated glia [93], CNS-infiltrating macrophages [94], and damaged neurons [13] acting as a potential cell death signal after interacting with TRAIL receptor-expressing cells resident in the CNS [92, 95–97].

An interesting aspect of TRAIL is its involvement in the homeostatic regulation of the immune system, as in fact, it is expressed on various innate and adaptive immune cell types [98], including monocytes, macrophages, natural killer cells, natural killer T cells, dendritic cells, and neutrophils after stimulation with lipopolysaccharide and pro-inflammatory cytokines such as IFNα, IFNβ, and IFNγ, as well as on T lymphocytes following T cell receptor (TCR)-mediated activation [99–104]. In contrast to the ligand, TRAIL-receptors are ubiquitously expressed also outside the immune system [105], and, for this reason, TRAIL seems to substantially modulate both the immune responses and their cellular components via the apoptotic cell-death pathway [106], and to participate to the immune response in different tissues and conditions [107–110].

Nevertheless, the immune system employs apoptosis not only as a self-restricting regulatory mechanism but also as an effector mechanism of immune-competent cells which can selectively eliminate virus-infected, transformed cells [111], and also normal cells in case of autoimmune inflammation [97], or in post-transplantion disorders [112]. TRAIL also represents an effector of immune-surveillance function and contribute to apoptosis of tumor and virus-infected cells [84].

TRAIL-induced apoptosis is involved in various processes, such as removal of lymphocytes with dangerous self-reactive specificities (autoreactive T and B cells) [113] and infiltrating immune cells [114, 115]. Data from studies carried out on TRAIL-deficient animal model suggest that TRAIL is essential for the maintenance of central immune tolerance by an indirect negative selection of autoreactive thymocytes [106, 116]. Additionally, TRAIL is involved in the regulation of peripheral tolerance by apoptosis-induction in mature lymphocytes after sensitization with IL-2, as well as by promoting the proliferation of Treg cells, elements with an essential role in maintaining immune tolerance [117, 118]. TRAIL increases anti-inflammatory Treg cell population as demonstrated by recent in vivo studies with systemically administered long-acting PEGylated TRAIL [119].

Moreover, TRAIL affects immune cells not only by inducing apoptotic death, but also by inhibiting their activation and expansion [120] as it directly inhibits T cell activation, suppresses T cell proliferation, and production of T cell-derived proinflammatory cytokines [108].

Finally, TRAIL system regulates innate and adaptive immune responses playing a role of crucial relevance in autoimmune and inflammatory diseases [11, 121, 122], as well as in immune surveillance in virtually all tissue and organs, including the CNS [123].

### TRAIL: a conductor of the inflammatory/immune orchestra in Alzheimer’s disease?

AD represents one of the greatest future global healthcare challenges. Owing to the increasing life expectancy in the general population and the consequent rising AD prevalence, this widely diffused disorder has become a major concern [124].

Neuropathologically, AD is characterized by the presence of amyloid plaques in the brain, as well as intracellular NFTs generated by hyperphosphorylated forms of the protein tau [2, 125], and in addition, by an inflammatory/immune response susceptibility, which plays a major role in various phases of the disease from its onset to later, progressive stages [21, 126].

It is noteworthy that neuroinflammatory foci in the AD brain localize in close vicinity of Aβ plaques, and they are associated with glia activation [127] and release of mediators of the inflammatory/immune response [128], including, among others, pro-inflammatory cytokines [129].

In this regard, TRAIL with its prominent death signaling and potent immune modulating properties [11] assumes an orchestrating role in the complex scenario of the AD brain.

TRAIL, specifically expressed in the human AD brain [130], is abundantly released by human neural cells challenged with Aβ in vitro [13] and activated glia [131], and is also associated with reduced expression of the Na+-Ca2+ exchanger neuroprotective isoform NCX3, with a subsequent reduction of the energetic supply to neurons, in such manner providing redundant contribution to its potent proapoptotic effect in course of neurodegenerative process [132].

Growing evidence suggest that TRAIL has a relevant coordinating function in the inflammatory roundabouts of AD, while it also directly mediates Aβ-related neurotoxicity (Table 1) [13, 14]. In fact, it has been demonstrated that immunoneutralization of TRAIL is associated with rescue from death of human neuronal cells challenged in vitro with Aβ [13], and that blockade of the DR5 TRAIL-death receptor signaling with specific antibodies completely abrogates Aβ-induced neurotoxicity in both human neuronal cell lines and primary cortical neurons [133], suggesting a direct, Aβ-additive neurotoxic effect of TRAIL in the AD brain. Based on these findings, it has been demonstrated that TRAIL immunoneutralization resulted in functional improvement, reduced deposition of Aβ and dramatically decreased expression of immune/inflammatory mediators in a transgenic mouse model of AD which develops progressive, age-related, cognitive decline [17].

**Table 1:** Pathophysiological implications of TRAIL in Alzheimer’s disease

Alzheimer’s disease model	TRAIL-based treatment	Main findings	Reference
SH-SY5Y neuronal-like cells	rTRAIL TRAIL-neutralizing monoclonal antibody	TRAIL mediates Aβ-neurotoxicity in vitro	[13]
Human AD brain	/	TRAIL is specifically expressed in Alzheimer’s disease brain	[130]
SH-SY5Y neuronal-like cells Primary mouse cortical neurons	anti-TRAIL-R/DR5 antibody	Blockade of TRAIL-death receptor DR5 signaling prevents Aβ-neurotoxicity in vitro	[133]
3xTgAD	TRAIL-neutralizing monoclonal antibody	Neutralization of TRAIL is associated with functional recovery, decreased Aβ burden and rebalance of both central and peripheral immune response in vivo.	[17]
3xTgAD	TRAIL-neutralizing monoclonal antibody	Neutralization of TRAIL restrain peripheral and CNS inflammatory/immune response along with decreased microglial TNFα production, reduced accumulation of both Aβ and p-Tau protein in the hippocampus of 3xTg-AD mice.	[18]

Summary of the most interesting evidences of the involvement of TRAIL in the pathophysiological events related to neuroinflammatory conditions such as Alzheimer’s disease, in view of a potential future clinical development of TRAIL-based therapeutic strategies

We have previously mentioned how misfolded proteins, such as Aβ, when not adequately removed, may drain into peripheral lymphoid organs, setting into motion and chronically maintaining an immune response [134], which, in turn, can result unbalanced in its outcome. In light of the fact that increased exchange of immunocytes may occur between peripheral lymphoid organs and the brain [135], it is noteworthy that the integrity of the BBB may not necessarily subsists in course of neurodegenerative disorders [136]. In fact, peripheral immunocytes have been indicated as factors that, when the inflammatory/immune equilibrium within the CNS is disrupted, are able to significantly influence progression of the AD brain pathology [74, 137].

Now, considering the pleiotropic role of TRAIL in orchestrating key events of the inflammatory/immune response, it appears of interest how its immunoneutralization also leads to a rebalance of immunocytes ratios, with special regard to the Treg cell population either in the spleen and in the brain [18].

Treg cells, besides their role as “controllers” of the overshooting inflammatory/immune response [138], when not represented in an adequate number, may be hired as causative elements of either hyperinflammatory [139] or proliferative [140] disorders. Thus, it is plausible to hypothesize that Treg cells represent, in a first phase of the response, a key factor in preventing fast progression of overshooting brain inflammation and consequent accelerated neurodegeneration, as a fruit of the adjustment of Treg (and, perhaps, of other immunocytes) flow to the brain, paralleled by decreased amount of Aβ and blunted immune reactivity.

Apparently, after a first attempt of the immune response to restain AD-related brain inflammation by means of TRAIL-driven increase of Treg cells, the latter may assume an overwhelming attitude, thus, limiting the beneficial effects of the immune response against accumulating Aβ [74], allowing the inflammatory response to overshoot, and resulting in noxious effects.

Consistently, central and peripheral immune/inflammatory markers, including specific Treg cells markers FoxP3 and GITR, as well as cyclooxygenase-2 (COX-2), inducible nitric oxide synthase (iNOS), IL-1β and TNFα are restored to basal levels, while, on the other hand, expression of anti-inflammatory cytokines such as IL-10 is significantly upregulated after chronic treatment of transgenic AD mice with anti-TRAIL antibody [18].

Moreover, such TRAIL-related restrain of peripheral and CNS inflammatory/immune response in murine model of AD occurs along with decreased both microglial TNFα production, along with reduced accumulation of both Aβ and p-Tau protein in the hippocampus of 3xTg-AD mice treated with an anti-TRAIL antibody [18].

## Conclusions

Redundant, persistent, and self-activating inflammatory processes in the brain undoubtedly represent one main factor fueling the progression of AD.

The concept of a dynamic, balanced modulation of the inflammatory/immune response has a relevant strength that should be exploited for discovery of innovative therapeutic strategies.

The pleiotropic effects of TRAIL appear evident within different outcomes of the inflammatory immune/response, consistently, either in the peripheral lymphoid organs and in the brain. The TRAIL system greatly influences neuronal death rate during neurodegeneration. Secondly, TRAIL also appears to be a connector of peripheral immune response with the degenerating inflamed brain, leading to activation of Treg cells and probably driving them to over-respond with detrimental consequences for the AD brain.

In conclusion, it is plausible to hypothesize that clinically meaningful treatment options for AD could be achieved through pharmacological modulation of the TRAIL system.

## Data Availability

Not applicable.

## Abbreviations

Aβ: Amyloid-beta
AD: Alzheimer’s disease
APCs: Antigen presenting cells
BBB: Blood-brain barrier
BCSFB: Blood-cerebrospinal fluid barrier
C1q: Complement component 1q
CNS: Central nervous system
COX-2: Cyclooxygenase-2
CP: Choroid plexus
CTLs: Cytotoxic T lymphocytes
DcRs: Decoy receptors
DR4: Death receptor 4
DR5: Death receptor 5
IFN-γ: Interferon gamma
IL-10: Interlekin-10
IL-1α: Interleukin-1alpha
IL-1β: Interleukin-1beta
IL-6: Interleukin-6
iNOS: Inducible nitric oxide synthase
MHC: Major histocompatibility complex
NFT: Neurofibrillary tangles
OPG: Osteoprotegerin
RNS: Reactive nitrogen species
ROS: Reactive oxygen species
TGFβ: Transforming growth factor beta
Th1: T-helper1 cells
Th17: T-helper17 cells
Th2: T-helper2 cells
TLR4: Toll-like receptors 4
TNF: Tumor necrosis factor
TNFSF10: TNF Superfamily Member 10
TNFα: Tumor necrosis factor alpha
TRAIL: TNF-related apoptosis-inducing ligand
Treg: Regulatory T cells
rTRAIL: Recombinant TRAIL
